# Design and optimization of localized plasmon resonance sensing via square-slotted Ag–graphene–dielectric metasurfaces for dermatological cancer identification using machine learning

**DOI:** 10.1038/s41598-025-28279-w

**Published:** 2025-11-21

**Authors:** Meshari Alsharari, Aymen Flah, Khaled Aliqab, Ivo Pergl, Abhinav Kumar, Ammar Armghan

**Affiliations:** 1 Department of Electrical Engineering, College of Engineering, Jouf University, 72388 Sakaka, Saudi Arabia; 2 College of Engineering, University of Business and Technology (UBT), Jeddah, 21448 Saudi Arabia; 3 Applied Science Research Center, Applied Science Private University, Amman, 11931 Jordan; 4 ENET Centre, CEET, VSB-Technical University of Ostrava, Ostrava, Czech Republic; 5 Department of Mathematical Sciences, Saveetha School of Engineering, SIMATS, Chennai, 602105 India Tamilnadu; 6 Department of Mechanical Engineering and Renewable Energy, Technical Engineering College, The Islamic University, Najaf, Iraq

**Keywords:** Surface plasmon resonance, Graphene, Ag, Biosensor, Metasurface, Engineering, Materials science

## Abstract

Skin cancer is a dangerous, life-threatening illness impacting countless individuals globally, requiring urgent awareness, prevention, and early detection. It is one of the most common forms of cancer, often caused by excessive sun exposure or tanning, and requires early detection for effective treatment. Early detection of skin cancer is achievable through advanced sensor designs that utilize graphene material. Graphene’s exceptional properties make it extremely appropriate for creating sensitive, accurate, and non-invasive diagnostic tools to identify cancer at early stages. The integration of silver (Ag), graphene, and silicon dioxide (SiO₂) materials forms a highly sensitive multilayer structure, significantly enhancing the surface plasmon resonance response, which enables precise detection of skin cancer biomarkers at extremely low concentrations. An exceptional sensitivity of 1050 nm/RIU is attained, enabling efficient skin cancer detection through advanced plasmonic biosensing technology. Optimizing the biosensor design by systematically varying key physical parameters—such as layer thicknesses, slot dimensions, and material configurations—significantly enhanced its sensitivity. The optimization is also achieved by using a Machine learning algorithm. The highest R^2^ value of 0.99 is achieved for this research. This strategic tuning of the structural and optical characteristics enabled more accurate detection capabilities, making the sensor highly effective for early skin cancer diagnosis through plasmonic resonance.

## Introduction

Skin cancer arises when abnormal skin cells grow uncontrollably, typically triggered by overexposure to ultraviolet (UV) radiation from sunlight or artificial tanning sources. As the most prevalent cancer type worldwide, it encompasses several forms. While often preventable through protective measures like sunscreen and avoiding excessive sun, early diagnosis remains critical. Detecting skin cancer at an initial stage significantly enhances treatment outcomes and long-term survival. Regular skin checks, public education, and increased awareness are essential tools in reducing risk and promoting healthier habits to combat this widespread yet often overlooked disease^1^. Early detection of skin cancer plays a vital role in significantly reducing the number of fatalities associated with the disease. By identifying skin cancer in its initial stages, individuals have a much higher chance of successful treatment and full recovery^2^. Skin cancer, if diagnosed early, is often less invasive and can be treated with less aggressive medical intervention. Delayed diagnosis, on the other hand, can allow the cancer to spread, leading to more complicated treatment procedures and a lower survival rate. Prompt medical consultations are essential components of an effective prevention strategy^3^. Dermatological screenings, self-checks for new or evolving moles, and public education campaigns can contribute to catching the disease before it becomes life-threatening. Encouraging early medical attention for suspicious lesions by minimizing the need for extensive treatment^4^.

Biosensors have emerged as a powerful device in the fast and early detection of cancer, proposing a highly efficient and non-invasive approach for identifying abnormal cells^5^. These advanced devices are designed to recognize specific biological markers associated with cancerous changes in the skin, allowing for precise diagnosis at an early stage. By converting biological responses into measurable signals, biosensors enable healthcare professionals to detect malignancies before they progress to more advanced and potentially life-threatening stages. Their portability, sensitivity, and ability to deliver real-time results make them particularly valuable in both clinical and remote settings^6^. Early diagnosis through biosensor technology significantly improves the chances of successful treatment and reduces the need for invasive procedures. Moreover, these tools are instrumental in continuous monitoring, offering patients an opportunity to track skin health regularly. As research advances, biosensors continue to evolve, becoming even more accurate, accessible, and affordable for widespread use in dermatological care and cancer prevention strategies^7^. Biosensors are designed for sensing different biomolecules. The biosensors are designed for sensing hemoglobin, and such biosensors are designed on a chip using research^8^. Sensors can also be designed to sense tuberculosis with the help of optimization and high sensitivity^9^. Ring resonators are also used to detect the sensing biomolecules in the sensor design^10^. Plasmonic sensing can be used for the detection of life-threatening diseases like cancer, diabetes, etc^11^. The blood biomolecules can be detected effectively with high efficiency using the biosensor designed with 2D materials^12^. Plasmonic biosensors can also be designed to detect different liquids for chemical sensing^13^. Cancerous cells can be effectively detected using a SPR-based biosensor design. The Jurkat cancer cell and PC 12 cancer cells have been effectively observed^14^. The biosensors can be implemented using a lab-on-chip approach and can be used for many applications^15^. Biosensors can also be designed with high sensitivity for different applications such as temperature^16^, virus^17^, pressure^18^, etc.

Various kinds of biosensors are employed for the recognition of cancer, each offering unique mechanisms and advantages to enhance diagnostic accuracy and efficiency^19^. Optical biosensors, for instance, use light-based techniques to detect molecular interactions, while electrochemical biosensors measure electrical signals resulting from biochemical reactions. Additionally, thermal and piezoelectric biosensors detect changes in temperature or mass, respectively, which may occur due to abnormal cell activity^20^. The integration of nanotechnology has further improved the sensitivity of these devices, enabling the recognition of cancer at very early stages. The use of different biosensors allows clinicians to select the most suitable tool based on the location and difficulty of the suspected skin cancer cells. Overall, the diversity of biosensor technologies plays a critical role in improving diagnosis^21^.

Graphene, a remarkable nanomaterial that is increasingly being utilized with extreme sensitivity to biochemical changes, makes it an ideal candidate for biosensor development^22^. Graphene-based biosensors can detect minute concentrations of cancer biomarkers, enabling the identification of skin cancer at a very early stage, often before visible symptoms appear. These sensors work by capturing and analyzing molecular interactions that indicate the presence of malignant cells. Additionally, graphene’s flexibility and biocompatibility make it suitable for wearable diagnostic devices that can monitor skin health in real-time^23^. Ongoing research continues to refine these technologies, aiming to improve their precision, affordability, and ease of use. The integration of graphene into skin cancer diagnostics represents a promising advancement in non-invasive, rapid, and highly accurate early detection methods, ultimately contributing to improved patient survival rates. The incorporating circular resonators in the improves the efficiency. These resonators enhance the sensor’s sensitivity by improving electromagnetic field confinement, allowing precise identification of cancer biomarkers^24^. Graphene is employed to achieve high sensitivity in detection systems. Their excellent response enhances signal results^25^. Graphene-based materials are utilized in sensing applications to enhance detection capabilities. Their strong fluorescence make them ideal for identifying biomarkers with great sensitivity. These features allow for improved accuracy and efficiency in detecting diseases such as skin cancer at early stages^26^.

Metamaterials are unique materials that help improve the sensitivity. These materials are now being employed to enhance the sensitivity of sensors used for the early and rapid detection of skin cancer^27^. By manipulating the behavior of electromagnetic waves, metamaterials can amplify the interaction between the sensor and biological samples, allowing for more precise detection of cancer-specific biomarkers^28^. This heightened sensitivity is crucial for identifying cancerous cells at the very beginning of their development, often before visible symptoms occur. Metamaterial-based sensors can operate across various frequency ranges, including optical and terahertz, which are particularly effective in medical imaging and diagnostics. Their ability to improve signal resolution and reduce background noise ensures faster and more reliable detection results. As a result, the incorporation of metamaterials into diagnostic technology is revolutionizing early skin cancer screening, making it more accurate, efficient, and potentially more accessible for widespread clinical and personal healthcare use. Metamaterials are increasingly being used in sensing technologies to detect skin cancer with enhanced precision. Their unique ability to manipulate electromagnetic waves enables improved signal control and sensitivity in diagnostic devices. By enhancing the interaction between the sensor and biological tissues, metamaterials’ efficient design contributes to faster treatment decisions and better patient outcomes through non-invasive and reliable skin cancer detection methods^29^.

The reviewed research highlights the need for a highly sensitive design to aid in skin cancer detection. To address this, a biosensor with enhanced sensitivity has been designed using graphene, known for its exceptional optical and electronic properties. Additionally, optimization has been implemented to further enhance the design’s performance, ensuring faster and earlier detection. The subsequent sections outline the design, modeling, and analysis of the graphene sensor. These steps include detailed examinations of the sensor’s structure, performance, and efficiency, along with results from simulations and experiments. The aim is to provide insights into its functionality and effectiveness in various applications and environments.

## Design analysis

**Fig. 1:**
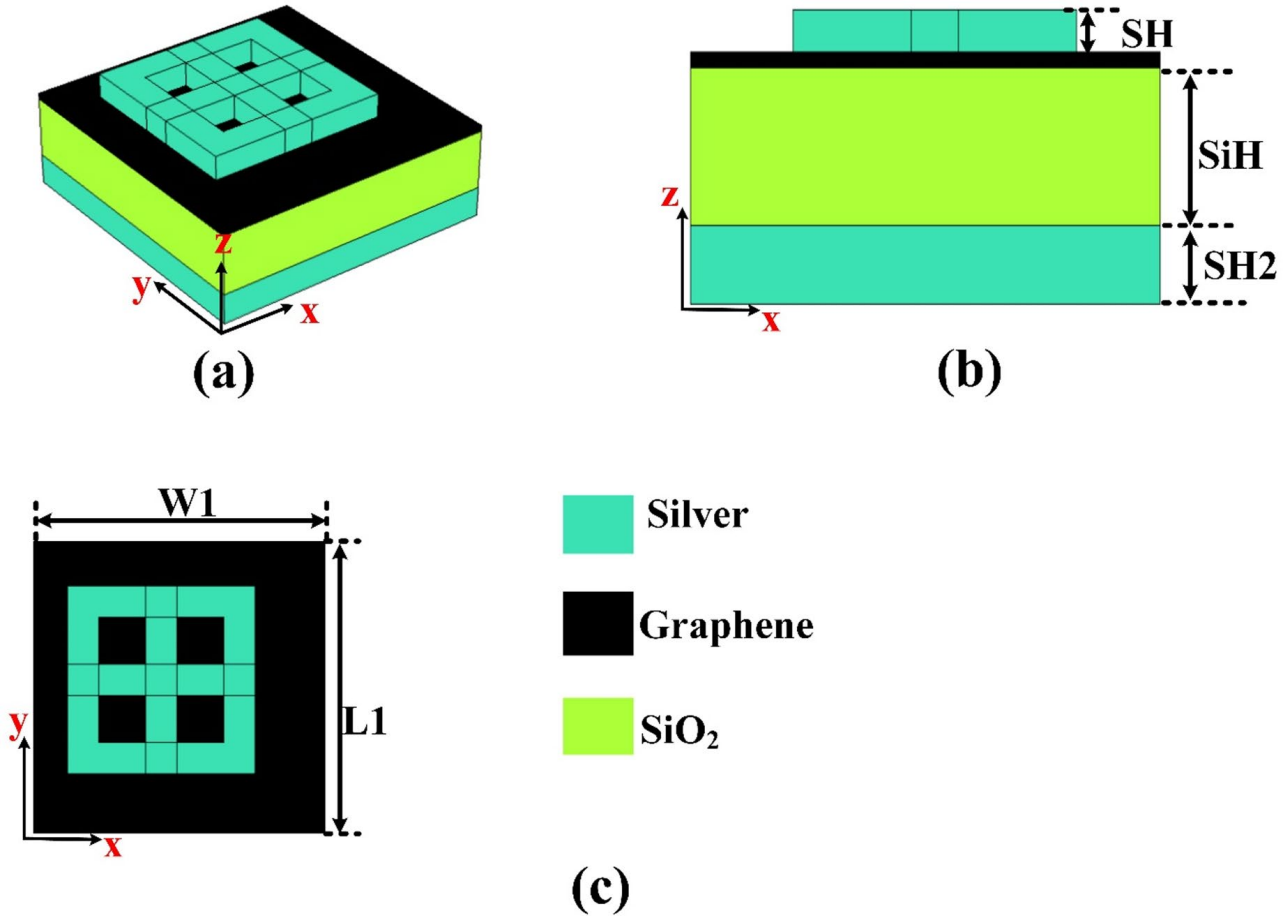
(**a**–**c**) Sensor design with its three different views and materials.

A graphene-based sensor has been carefully designed to achieve high sensitivity for skin cancer detection. This innovative sensor utilizes the unique electrical and optical properties of graphene to enable precise identification of cancer-related biomarkers^30^. The structural configuration of the design gives a better performance and responsiveness. Its design with three different views of the sensor—top, side, and perspective—is illustrated in Fig. 1. These views offer detailed visual representations of the sensor’s layout and construction, highlighting the key features and dimensions that contribute to its effective functioning in biosensing applications.

The sensor design incorporates a Metal-Insulator-Metal (MIM) structure, utilizing a combination of silver (Ag), silicon dioxide (SiO₂), and silver (Ag) layers. This configuration enhances optical and plasmonic properties, which are critical in sensor design. In this MIM structure, silver serves as the reflective base, while the middle layer of SiO₂ serves as the insulating dielectric layer. The top silver layer functions as the active sensing surface, interacting with incident light and target biomolecules.

The integration of these specific materials is deliberate—silver is known for its excellent plasmonic behavior, which enables strong surface plasmon resonance (SPR) effects, while silicon dioxide offers stability, transparency, and compatibility with various fabrication processes. The MIM configuration allows for effective confinement and manipulation of electromagnetic fields within the sensor, thereby significantly boosting its detection capabilities. By precisely controlling the thickness and arrangement of each layer, the sensor can be optimized for maximum sensitivity and selectivity toward specific skin cancer biomarkers. This layered design not only enhances performance but also provides overall stability for application in medical diagnostics.

The fabrication of MIM design, with overall performance with functionality of a biosensor. In this process, the MIM layers—comprising silver and silicon dioxide are sequentially deposited onto the substrate using standard deposition. Precise control over the thickness of each layer is maintained to ensure optimal optical and plasmonic behaviour.

**Fig. 2:**
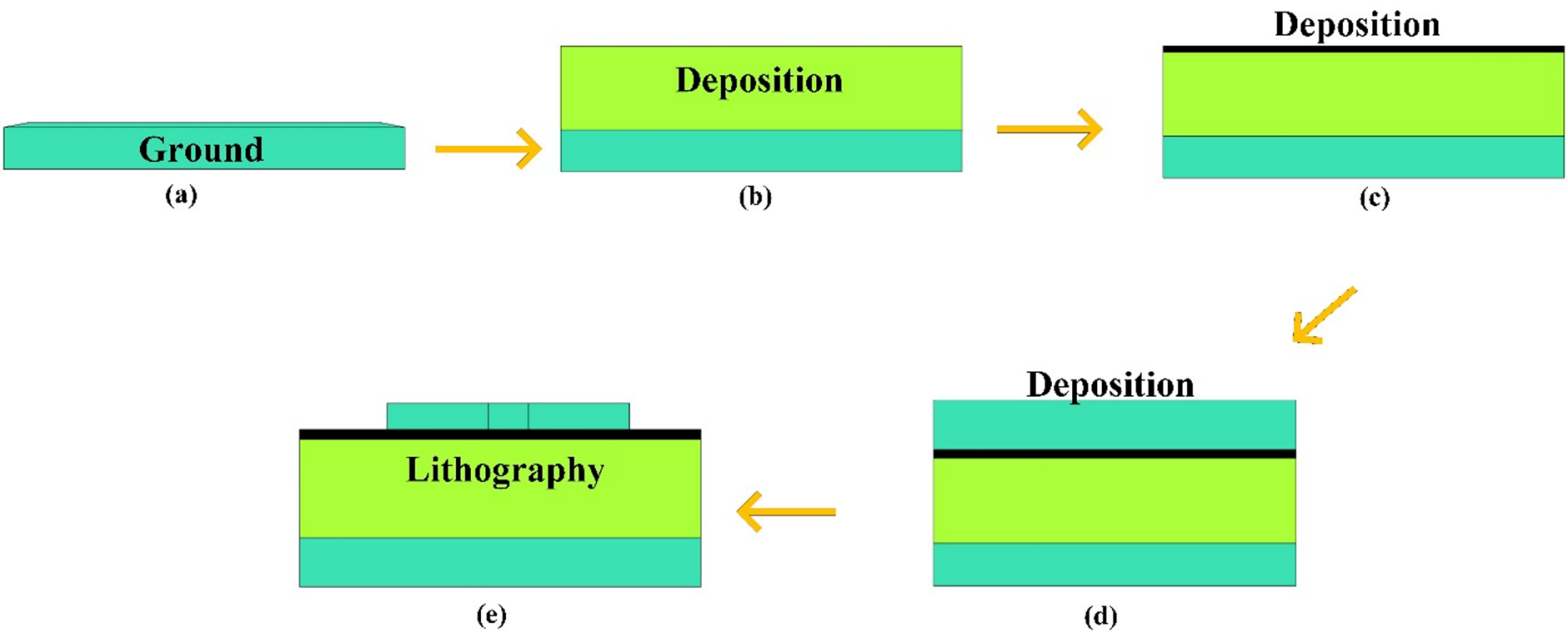
(**a**–**e**) Fabrication approach of the multilayer design with deposition of layers and lithography.

Following the deposition, advanced lithography techniques are employed to pattern the desired resonator structure. In particular, a square-slotted resonator shape is created to improve performance. This is achieved through photolithography or electron beam lithography, where a specific pattern is developed to mark the areas to be etched. Subsequently, etching methods are used to remove material from the exposed regions, forming the intricate resonator geometry. The resulting structure, as illustrated in Fig. 2, supports strong localized surface plasmon resonances (LSPR), which significantly enhance the performance.

In this research, graphene is utilized as a sandwiched layer within the sensor structure, playing a critical role in enhancing the device’s performance. Owing to its remarkable performance, graphene is particularly effective in boosting sensitivity. One of the key characteristics that makes graphene suitable for such applications is its tunable conductivity, which significantly influences. The electrical conductivity of graphene is not fixed; instead, it varies depending on the graphene’s potential, which can be adjusted externally through gating or chemical doping. The tuning gives the sensor optimization for different operational frequencies and enhances the resonance response of the system. The mathematically modeled to accurately predict and control its behavior in the sensor. These relationships are described in detail through Eqs. (1–4)^31^. This provides the theoretical foundation for calculating the conductivity under various conditions. By incorporating graphene in a sandwiched configuration and leveraging its variable conductivity, the sensor achieves superior field confinement and improved signal detection. This approach is vital with tunable biosensors for reliable skin cancer diagnosis^32^.

1 $$\:\epsilon\:\:\left(\omega\:\right)=1+\:\frac{{\sigma\:}_{s}}{{\epsilon\:}_{0}\omega\:\nabla\:}$$

2 $$\sigma _{{\mathrm{int} ra}} = ~\frac{{ - je^{2} k_{B} T}}{{\pi \hbar ^{2} ~\left( {\omega - j2} \right)}}\left( {\frac{{\mu _{c} }}{{k_{B} T}} + 2~\ln \left( {e^{{\frac{{\mu _{c} }}{{k_{B} T}}}} + 1} \right)} \right)$$

3 $$\sigma _{{inetr}} = ~\frac{{ - je^{2} }}{{4\pi \hbar ~}}\ln \left( {\frac{{2\left| {\mu _{c} } \right| - \left( {\omega - j2} \right)\hbar }}{{2\left| {\mu _{c} } \right| + \left( {\omega - j2} \right)\hbar }}} \right)~$$

4 $$\:{\sigma\:}_{s}={\sigma\:}_{intra}+{\sigma\:}_{inter}$$

Chemical Potential (*µ*_*c*_): Varied from 0.1 eV to 0.9 eV to simulate different doping levels.

Scattering Rate (*Γ*): Fixed at 0.1 meV, corresponding to high-quality graphene at room temperature.

Temperature (*T*): Set at 300 K (room temperature), unless otherwise stated.

Reduced Planck’s Constant (*ħ*): Taken as 1.0545718 × 10^− 34^ J·s.

The modulation of µ_c was achieved via electrostatic gating, wherein an external bias voltage is applied across a dielectric layer beneath the graphene sheet. This enables dynamic tunability of the Fermi level, making the device suitable for reconfigurable sensor applications.

These parameters were consistently applied throughout all simulations to ensure accurate modeling of graphene’s frequency-dependent conductivity within the THz to infrared regime.

Equations (5–8) provides the analytical foundation for evaluating various key sensitivity parameters critical to the system’s performance^33^. In this context, four principal parameters are explored in depth: DL, FOM and Q-factor, and Sensitivity. The sensing mechanism under investigation. The Detection Limit refers to the minimum measurable change in the input signal that can be reliably distinguished by the system. A lower DL indicates enhanced sensitivity. The Figure of Merit combines sensitivity with resolution, serving as a comprehensive performance index that balances detection capability against spectral sharpness. A higher Q-factor signifies a narrower and more defined resonance peak, which typically translates to improved measurement accuracy. Sensitivity, in this context, is defined as the change in output per unit change in the measured parameter, indicating the system’s responsiveness. These parameters are systematically examined through theoretical analysis and simulation to assess their impact on sensor behavior, ensuring a thorough understanding of the device’s operational characteristics and performance potential.

5 $$S = \frac{{\Delta \lambda }}{{\Delta n}}$$

6 $$\:FOM\:=\:\frac{S}{FWHM}$$

7 $$\:Q=\frac{\lambda\:r}{FWHM}$$

8 $$\:DL=\:\left(\frac{\varDelta\:n}{1.5}\right)\times\:{\left(\frac{FWHM}{\varDelta\:\lambda\:}\right)}^{1.25}$$

## Results analysis

**Fig. 3:**
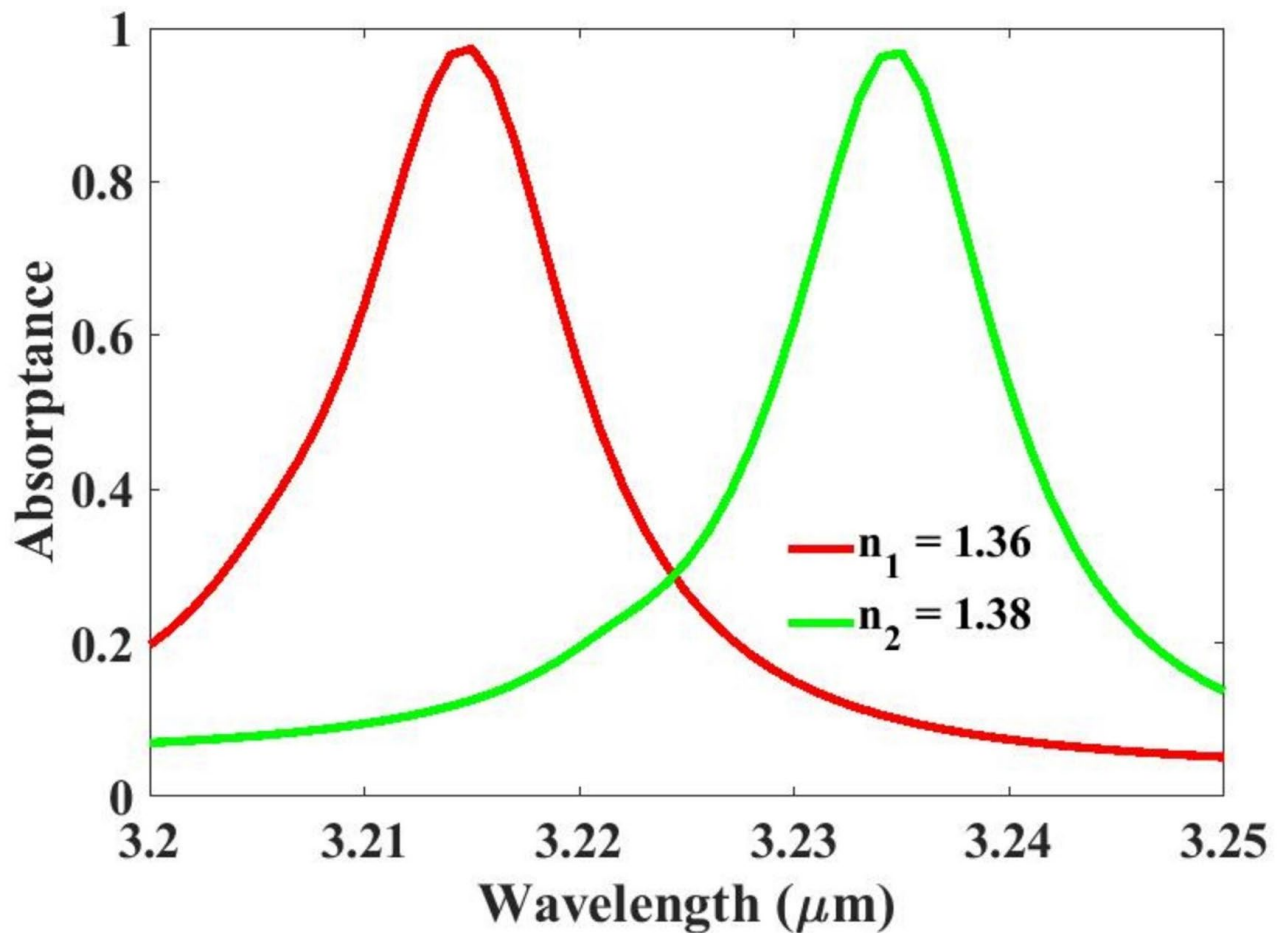
Sensitivity analysis using absorption results. The red color line shows the normal cell with a refractive index of 1.36, and the green color line shows the skin cancer cell with a refractive index of 1.38. The spectra are computed for TM-polarized incident light.

The analysis of simulation outcomes was conducted using COMSOL Multiphysics, a comprehensive platform for modeling and simulating physical systems. This software facilitated the evaluation of key results. We have used periodic boundary conditions, and port 1 and port 2 is applied on front and back. The design is polarization-insensitive for TE and TM polarizations. The Delaunay tessellation meshing is used in this research. By leveraging its advanced visualization and postprocessing tools, we were able to assess the system’s behavior under various conditions, ensuring accurate and reliable results. Figure 3 illustrates the sensitivity results derived from our proposed sensing structure, highlighting its ability to distinguish between different biological cells based on their refractive indices. The figure illustrates that the normal cell, exhibiting a refractive index of n₁ = 1.36, and the skin cancer cell, with a refractive index of n₂ = 1.38^24^, generate clearly distinct resonance peaks. This difference in refractive indices results in separate and identifiable resonance frequencies, allowing for the differentiation between the two cell types. The separation of these peaks provides valuable information for detecting and distinguishing normal cells from cancerous cells, contributing to more precise diagnostic techniques and advancing the understanding of cell behavior in various conditions. This clear spectral shift indicates that the sensor can accurately detect small variations in the optical properties of biological tissues.

The improved sensitivity of 1050 nm/RIU signifies a high level of responsiveness in detecting minute biological differences. This performance metric confirms the sensor’s effectiveness for biomedical diagnostic applications. In addition to sensitivity, other performance parameters were also evaluated. The Figure of Merit (FOM) was calculated as 105, reflecting a strong balance between sensitivity and spectral resolution. The Quality Factor (Q-factor) reached 321, indicating sharp and well-defined resonance peaks. Furthermore, the Detection Limit (DL) was found to be 0.00527 RIU, confirming the sensor’s precision and high detection capability for subtle refractive index changes.

The optimization of the simulation results has been further enhanced through the implementation of a parametric optimization approach. This method involves systematically varying and fine-tuning the physical and structural parameters of the sensing design in order to maximize its performance, particularly in terms of sensitivity. By adjusting key geometric dimensions—such as layer thicknesses, resonator widths, material refractive indices, and coupling gaps—a more refined configuration of the sensor is achieved. Through this iterative process, each parameter is evaluated for its influence on the sensor’s output response. The goal is to identify the optimal combination of values that lead to the highest possible sensitivity without compromising other critical performance indicators. The parametric study allows for detailed insight into how individual physical changes affect the resonance wavelength shift and the overall spectral response. This optimization not only ensures improved sensitivity, but also enhances the overall stability, resolution, and selectivity of the design. As a result, the final sensor configuration demonstrates superior performance characteristics.

**Fig. 4:**
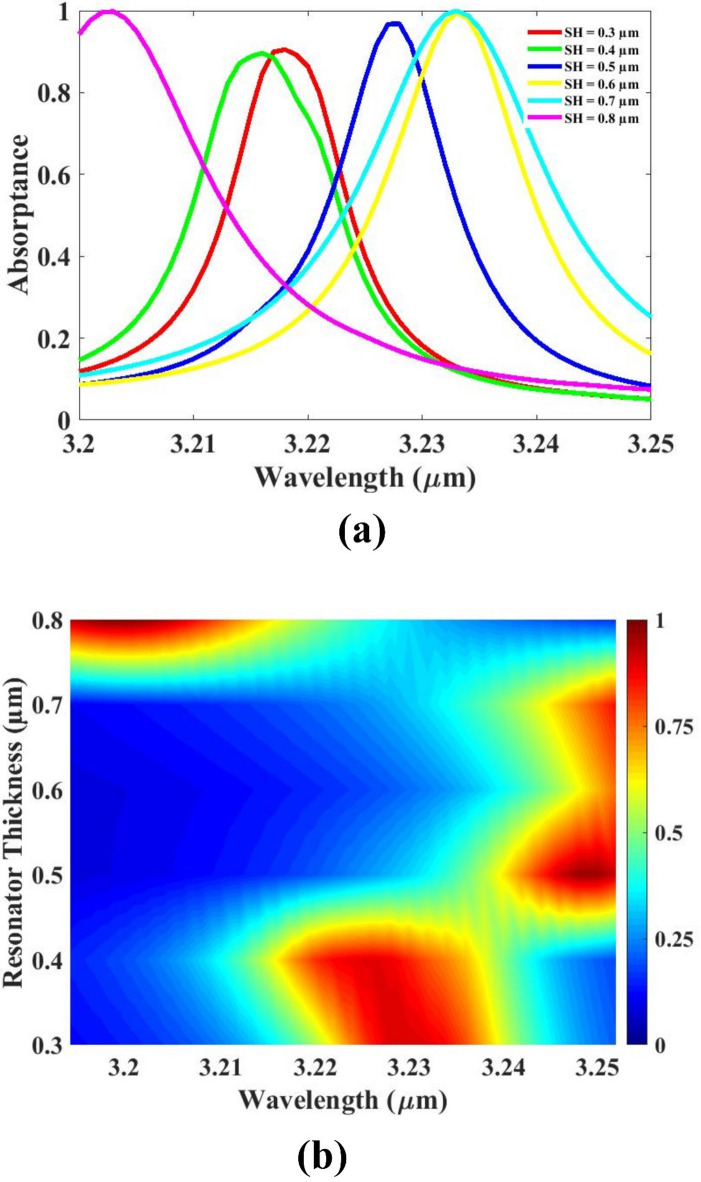
(**a**, **b**) Absorption for resonator thickness variation. Different colors indicate different thicknesses.

Figure 4 presents the detailed investigation of the sensor’s performance response with respect to variations in the resonator thickness. This analysis is carried out using both line plots and color plots to comprehensively visualize the effects of different thickness values on the sensor’s absorption characteristics. The primary objective of this parametric study is to determine the optimal thickness that yields the most favorable optical response for enhanced sensitivity and device efficiency. As shown in the plots, changing the resonator thickness leads to noticeable shifts in resonance behavior, including variations in peak absorption intensity and sharpness. The results indicate that a thickness of 0.5 μm offers the most effective configuration. At this optimized value, the sensor achieves a well-defined, sharp absorption peak, which is essential for precise detection. Additionally, this thickness provides strong absorption while maintaining a compact structural profile, which is desirable for practical sensor integration. This optimized thickness ensures that the sensor exhibits a high-quality spectral response, contributing to improved sensitivity and selectivity. The study confirms that precise control over physical parameters like resonator thickness.

**Fig. 5:**
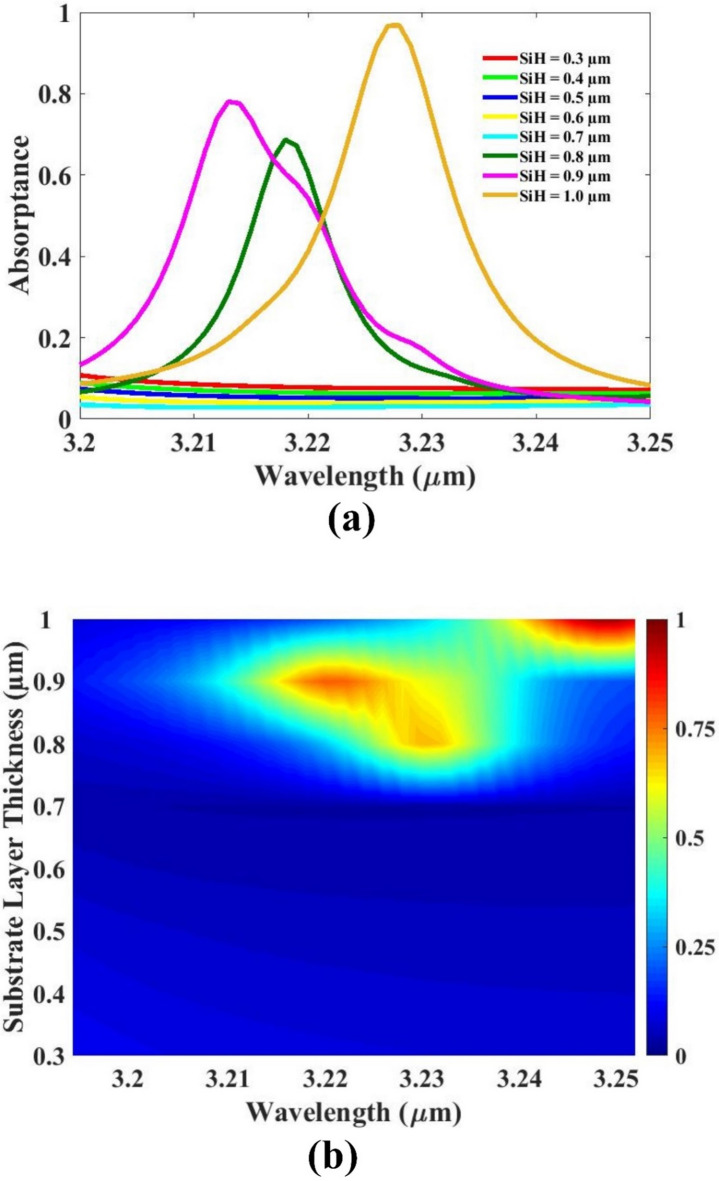
(**a**, **b**) Absorption for substrate thickness variation. Different colors indicate different thicknesses.

An additional parametric study was performed by varying the substrate thickness to observe its influence on the absorption response of the sensor. The results, illustrated in Fig. 5, provide a clear visual representation of how different substrate thicknesses impact the sensor’s optical performance, particularly in terms of absorption intensity. The analysis reveals that the absorption response is significantly affected by changes in substrate thickness. Among all tested values, a thickness of 1 μm consistently exhibits the highest absorption peak, indicating superior energy confinement and interaction within the sensing region. For all other thickness values—whether lower or higher than 1 μm—the absorption is notably reduced, suggesting less effective resonance behavior and weaker field enhancement. This finding highlights the critical importance of optimizing substrate thickness to ensure maximum absorption efficiency. Selecting the 1 μm thickness not only maximizes absorption but also contributes to sharper and more defined resonance peaks, which are essential for accurate and sensitive detection in practical sensing applications. Therefore, based on this investigation, a substrate thickness of 1 μm is chosen as the optimal value. This ensures enhanced optical performance, supporting the overall goal of achieving a high-performance sensor capable of reliable and precise detection across a range of applications.

**Fig. 6:**
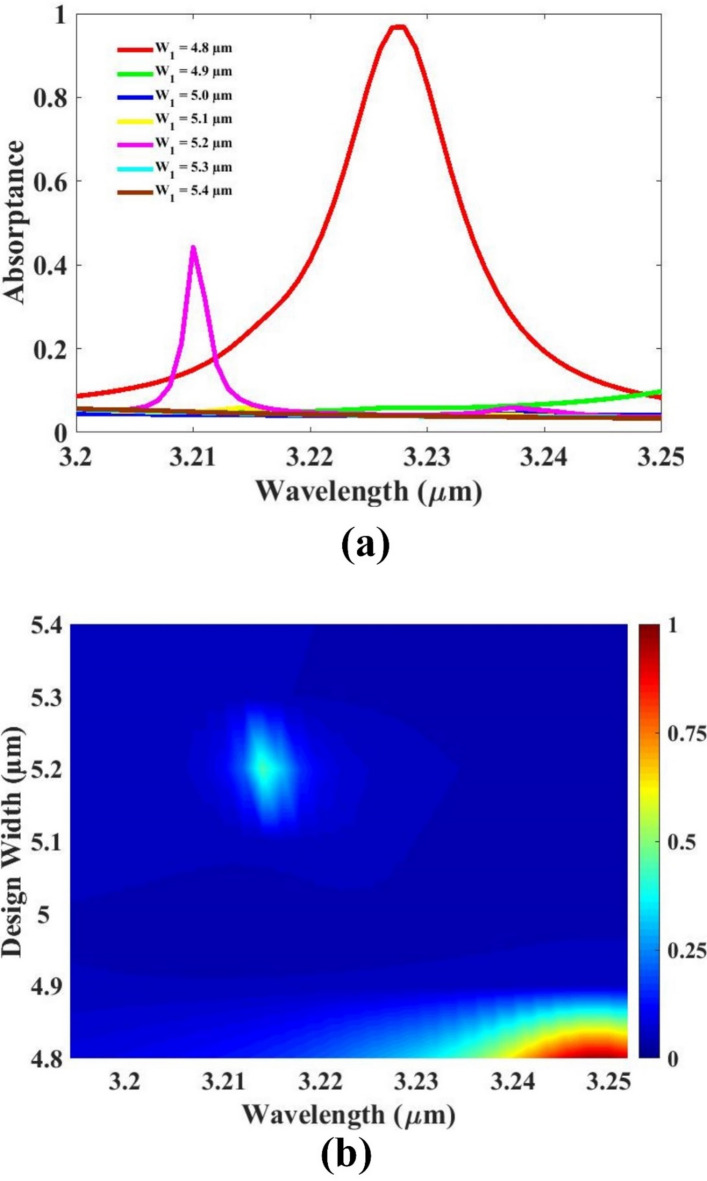
(**a**, **b**) Absorption for substrate width variation. Different colors indicate different widths.

The effect of width variation on the sensor’s absorption response is thoroughly analyzed and illustrated in Fig. 6. In this study, the width of the sensing structure is varied within the range of 4.8 μm to 5.4 μm, and the corresponding absorption characteristics are evaluated through line plots. Each plot is color-coded for clarity, with the response for 4.8 μm highlighted in red to emphasize its performance. The simulation results clearly indicate that the 4.8 μm width produces the highest absorption peak among all tested values. As the width increases beyond 4.8 μm, the absorption response decreases significantly, resulting in weaker resonance and reduced sensing capability. This demonstrates that narrower structural widths lead to better energy confinement and stronger interaction with the incident light, which are crucial for efficient sensor operation. In addition to its superior optical performance, the 4.8 μm width also offers practical advantages in terms of cost efficiency and compact device size. A smaller width reduces material usage and allows for more compact integration into sensing platforms. Based on these results, the optimized width is set at 4.8 μm, ensuring high absorption, enhanced sensitivity, and economic feasibility.

**Fig. 7:**
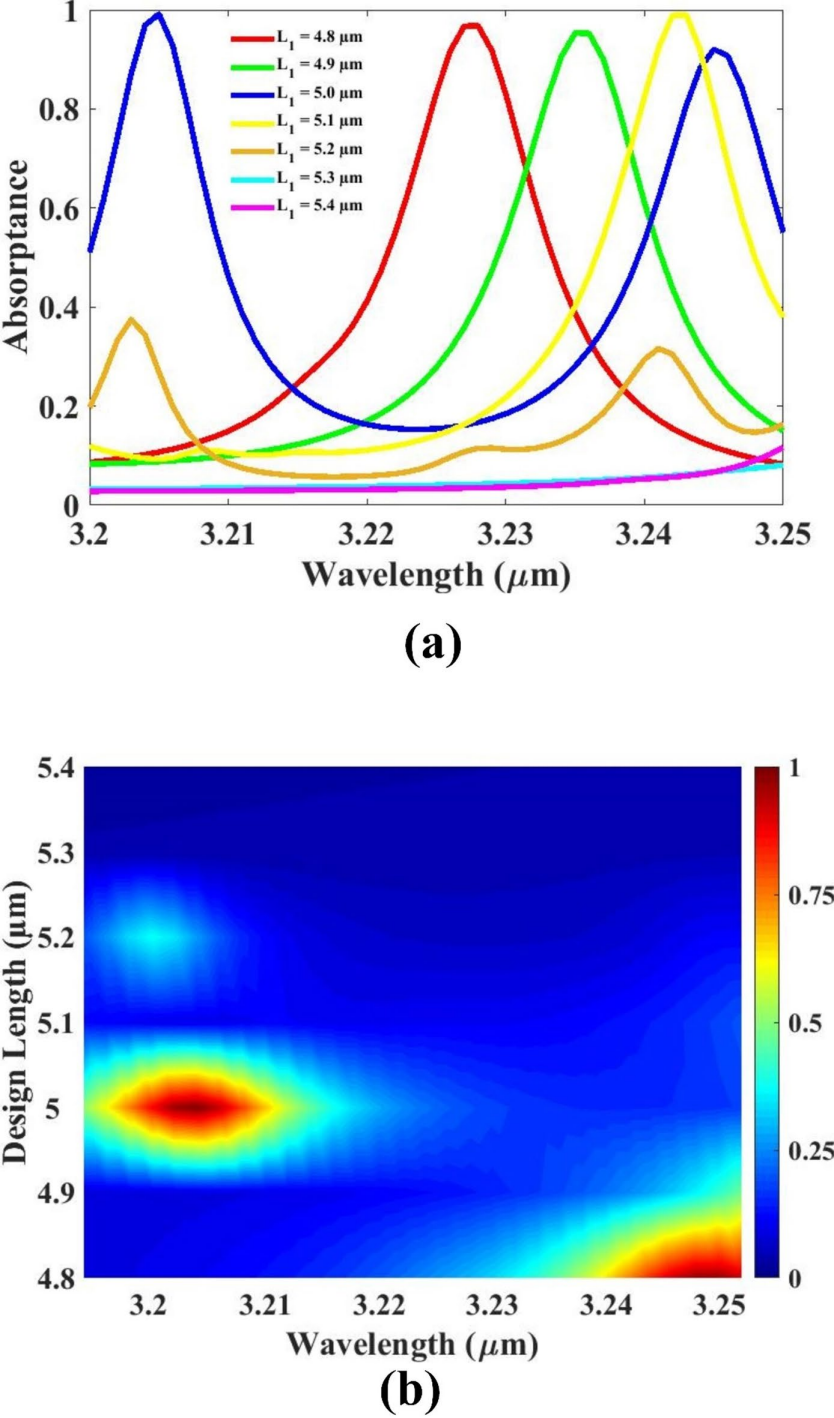
(**a**, **b**) Absorption for substrate length variation. Different colors indicate different lengths.

The impact of varying the structural length on the sensor’s absorption performance is analyzed and depicted in Fig. 7. In this study, the length is systematically adjusted from 4.8 μm to 5.4 μm, and the corresponding absorption responses are recorded to identify the most effective value for optimizing sensor performance. The results show that five different length values within this range demonstrate notably higher absorption peaks compared to the others. These peaks indicate strong resonance behavior and efficient light–matter interaction, both of which are essential for achieving high sensitivity in sensing applications. Among these five high-performing lengths, the lowest length value of 4.8 μm stands out as the most efficient and practical option. It delivers strong absorption while maintaining a compact device footprint. Selecting the 4.8 μm length not only ensures excellent optical response but also supports device miniaturization and cost-effectiveness, which are important for real-world applications requiring integrated, low-power sensors. Based on the comparative analysis, 4.8 μm is chosen as the optimized length value, balancing high absorption performance with physical compactness and design efficiency, thereby enhancing the overall utility and scalability of the proposed sensor design.

**Fig. 8:**
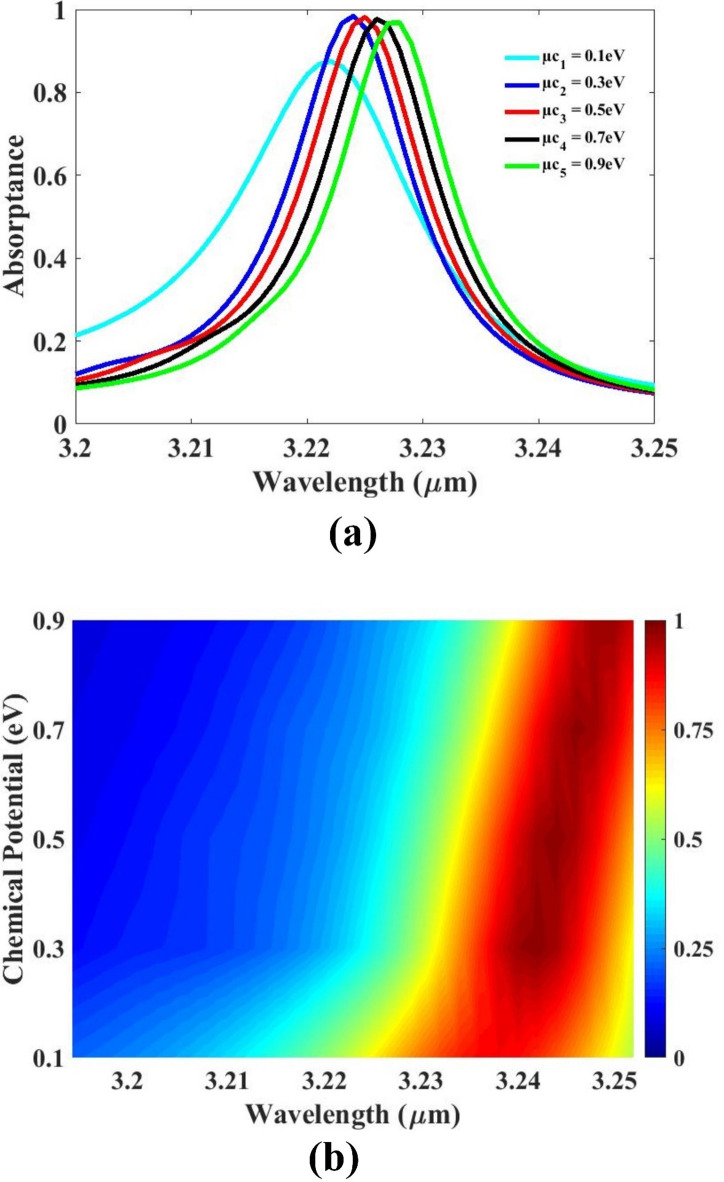
(**a**, **b**) Absorption for chemical potential variation. Different colors indicate different chemical potentials.

The effect of the sensor’s absorption response is thoroughly investigated and presented in Fig. 8. In this analysis, the graphene potential is systematically adjusted from 0.1 eV to 0.9 eV to study its influence on the optical properties of the sensor. Graphene, due to its unique electronic and optical characteristics, offers excellent tunability when integrated into photonic structures. The results clearly demonstrate that as the graphene potential is varied, noticeable tuning of the absorption spectra occurs. This spectral shift confirms that the sensor’s response can be actively controlled through the adjustment of graphene’s chemical potential, enabling dynamic modulation of resonance behavior. The ability to tune the sensor in real time adds significant value for applications that require adaptable performance across different environments or target materials. This tunable behavior introduces a new level of flexibility and functionality to the sensor design. By exploiting the adjustable electronic properties of graphene, the proposed sensor can be tailored for multi-parameter sensing or target-specific detection with enhanced precision. Based on these findings, the design incorporates graphene as a key material to realize a tunable, high-performance optical sensor with broad application potential.

**Fig. 9:**
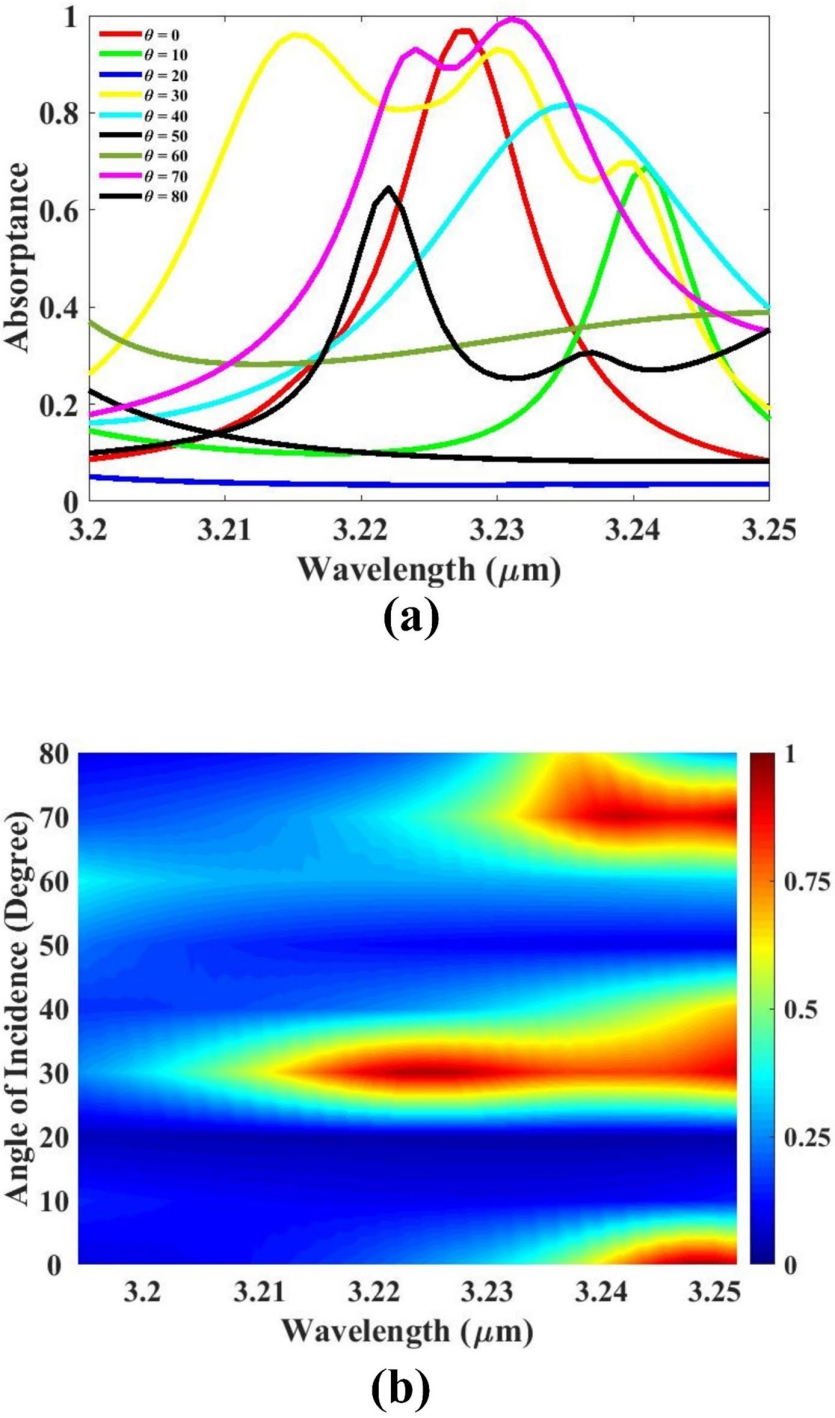
(**a**, **b**) Absorption for angle of incidence variation.

The influence of incident angle variation on the sensor’s absorption performance is systematically studied and the results are presented in Fig. 9. In this analysis, the angle of incoming light is varied from 0° to 80°, and the corresponding absorption response is evaluated to understand the angular sensitivity and robustness of the sensor design. The results clearly show that the absorption remains high and stable when the incident angle ranges from 0° to 40°. Within this interval, the sensor maintains strong resonance behavior and efficient light–matter interaction, which are critical for achieving high sensitivity and accurate detection. This angular stability is particularly valuable in practical sensing applications, where light may not always strike the sensor at a perfectly normal incidence. Beyond 40°, a noticeable decline in absorption is observed, indicating reduced interaction between the incoming electromagnetic wave and the sensing structure. Therefore, the optimal angular operating range is identified as 0° to 40°, ensuring both strong absorption and tolerance to moderate angular deviations. This angular analysis confirms that the proposed sensor is effective within a broad and practical range of incident angles, making it suitable for real-world applications where precise alignment may not always be possible.

**Fig. 10:**
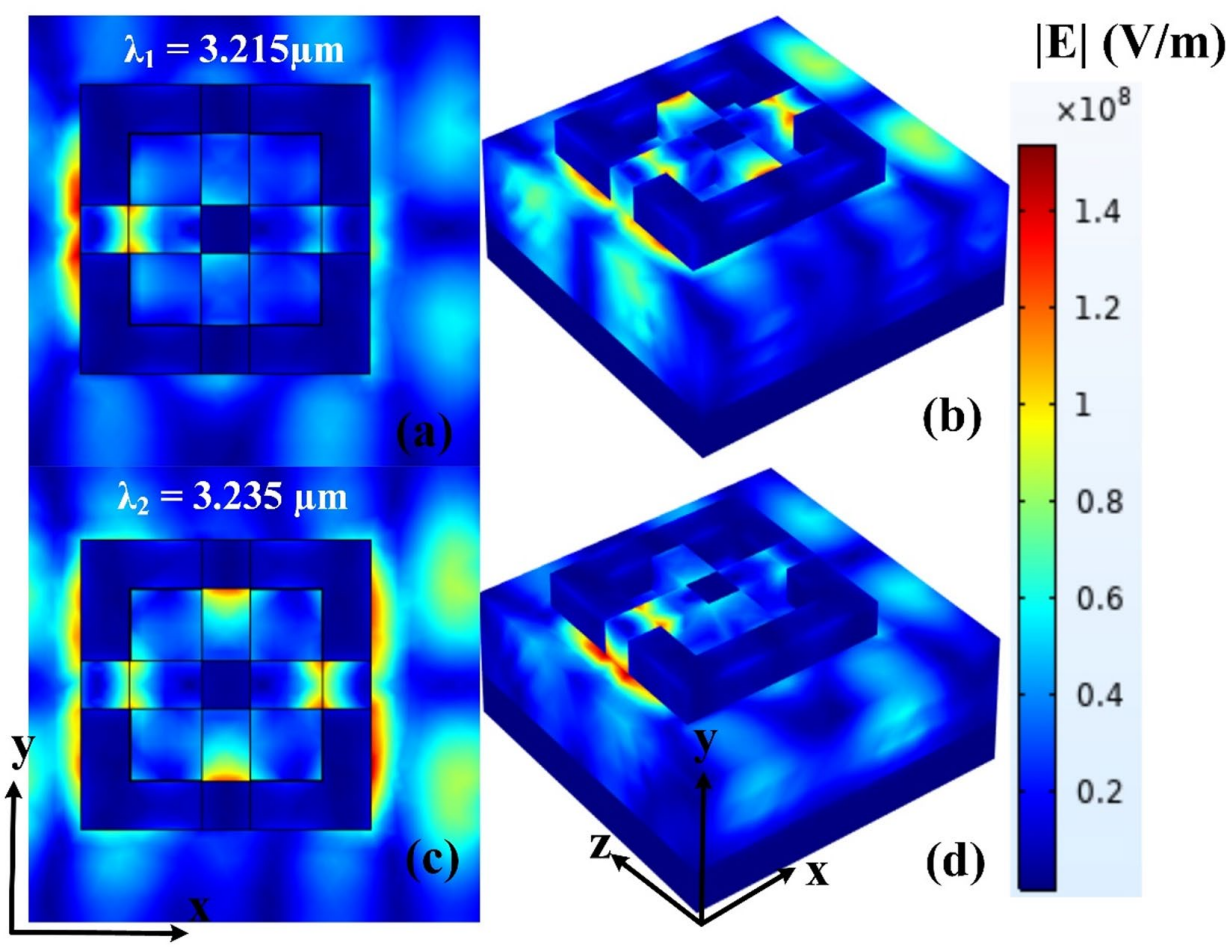
(**a**–**d**) The electric field response for two different wavelengths.

The electric field distribution of the proposed sensor design has been thoroughly analyzed at two distinct resonance wavelengths: 3.215 μm and 3.235 μm. The simulation results are visually represented in Fig. 10, showcasing both the top view and three-dimensional (3D) view of the electric field intensity within the structure. These visualizations provide deep insight into the interaction between the incident electromagnetic waves and the resonator geometry. At both wavelengths, the electric field is strongly confined within specific regions of the sensor, highlighting the effectiveness of the structure in supporting localized surface plasmon resonance or guided-mode resonance, depending on the design. The comparison between the two wavelengths shows a slight shift in field localization, which aligns with the resonant behavior expected from a finely tuned photonic structure. The top view offers a detailed look at the planar distribution of the field, revealing symmetrical and well-defined hotspots, while the 3D view illustrates the vertical confinement and intensity gradients more clearly. These results confirm that the sensor supports strong field enhancement at resonant wavelengths, which is crucial for achieving high sensitivity in practical sensing applications. This electric field analysis validates the structure’s ability to localize energy effectively for optimal sensing performance.

A comprehensive comparison of sensitivity-related parameters between our proposed design and several previously published designs is presented in Table 1. This comparative analysis highlights the effectiveness of our approach by evaluating key performance metrics such as Sensitivity, Detection Limit (DL), Figure of Merit (FOM), and Quality Factor (Q-factor). These critical indicators serve as benchmarks for assessing the operational superiority of optical or photonic sensing systems. Upon analyzing the data, it becomes evident that our design consistently outperforms existing solutions documented in the literature across all evaluated parameters. Notably, our system exhibits significantly higher sensitivity, indicating an enhanced ability to detect even minor changes in the measured quantity. Furthermore, our design demonstrates a lower detection limit, suggesting improved capability in identifying subtle variations. The figure of merit is also substantially higher, implying a more optimal trade-off between sensitivity and resolution. Additionally, the elevated Q-factor observed in our design points to a sharper resonance peak, which contributes to greater precision and selectivity. Overall, the results presented in Table 1 validate the superior performance of our design. The improvements across multiple performance metrics establish its potential as a highly efficient and reliable alternative to existing sensing technologies.

**Table 1:** Comparison.

Design	Sensitivity (nm/RIU)	Quality factor (Q)	Figure of merit (FOM) RIU^− 1^	Detection limit (DL) RIU
^34^	500	-	-	-
^35^	546.72	2066.44	-	1.44
^36^	1000	-	-	-
^37^	359	-	-	-
^38^	153	-	-	-
^39^	161	-	-	-
^40^	74.5	19.82	-	-
^32^	1100	-	3.832	0.391
^41^	387	-	-	-
^42^	690	2500	1400	-
^43^	900	190	105	0.00001
^44^	306.25	5000	103	-
^45^	349	-	-	-
^46^	1000	-	-	-
^47^	200	3493	222	-
^48^	1400	-	-	-
^49^	253	-	-	-
^50^	-	262	-	0.002
^51^	1000	5166	2.94	0.04
Cervical cancer sensor	1050	321	105	0.00527

## Conclusion

The proposed graphene-based optical sensor is innovatively designed using a hybrid combination of silver (Ag) and silicon dioxide (SiO₂) materials, selected for their superior plasmonic and dielectric properties, respectively. Silver provides strong surface plasmon resonance, enhancing light–matter interaction, while SiO₂ offers structural support and excellent optical transparency, making this combination ideal for advanced sensing applications. The sensor is specifically tailored to detect skin cancer cells by distinguishing subtle refractive index differences between healthy and malignant tissues. Comprehensive numerical simulations and parametric optimization techniques were employed to refine key design parameters—including the thickness, width, and length of the resonator and substrate layers. These optimizations ensure maximum electromagnetic field confinement and resonance enhancement, directly contributing to the sensor’s performance. The finalized design achieves an impressive sensitivity of 1050 nm/RIU, DL of 0.00527 RIU, Q-factor of 321 and FOM of 105 RIU^− 1^, indicating its high efficiency in detecting minute biological variations. In addition, the integration of graphene allows for electrical tunability, adding flexibility to the sensor’s functionality. The optimization is also achieved by using a Machine learning algorithm. The highest R^2^ value of 0.99 is achieved for this research. The compact size, high sensitivity, and tunable characteristics make the sensor an excellent candidate for non-invasive, real-time biomedical diagnostics, particularly for early detection of skin cancer. This work demonstrates the strong potential of nanophotonic designs in revolutionizing disease detection technologies.

## Data Availability

The data supporting the findings in this work are available from the corresponding author with reasonable request.
